# Letter to the Editor for the article ‘Pharmacologic prevention and therapy of postoperative paralytic ileus after gastrointestinal cancer surgery – systematic review and meta-analysis’

**DOI:** 10.1097/JS9.0000000000001566

**Published:** 2024-05-09

**Authors:** Yajie Wang, Shumin Gao, Yisheng Pan

**Affiliations:** a Department of Gastrointestinal Surgery, Peking University First Hospital; b National Institute on Drug Dependence, Peking University, Beijing, People’s Republic of China

*Dear Editor,*

We were interested in reading the impressive study by Reichert *et al*.^1^ in the *International Journal of Surgery* and would like to congratulate the authors for their interesting findings. Although evidence for pharmacological prevention of postoperative paralytic ileus (POI) following gastrointestinal tumor surgery is limited, authors included 55 RCTs (randomized controlled trials) in this huge meta-analysis, of which 5078 people received the experimental intervention and 3857 people experienced placebo, standard treatment, or no treatment. The author summarizes the approaches to preventing POI, including opioid-sparing analgesia, peripheral opioid antagonism, the reduction of sympathetic hyperreactivity, and early use of laxatives effectively, which is very useful for clinical practice. However, we wish to discuss the following concerns.

First, the authors limited their literature search to MEDLINE, CENTRAL, as well as information extracted from ClinicalTrials.gov. However, they did not explore other international databases with broad subject areas, including Embase, Cochrane Central Register of Controlled Trials, Web of Science, and Scopus databases. The selection of a restricted subset of databases for conducting the literature search can lead to biased results and conclusions^2^.

Second, according to our clinical practice and related studies^3-5^, compared with minimally invasive surgery (MIS), which includes laparoscopic-assisted surgery and robot-assisted surgery, open surgery (OS) significantly increases the probability of POI. Therefore, it is suggested that the authors conduct a subgroup analysis of different surgical methods (OS or MIS) to ensure more accurate results.

Third, we may think about the effect of abdominal surgery at different sites on the occurrence of postoperative POI and on prevention and treatment outcomes. Different parts of gastrointestinal motility disorders produce different clinical manifestations. For example, the main symptoms of gastric dysmotility are concentrated in nausea, vomiting, and upper abdominal distension, while the main symptoms of intestinal motility disorders are abdominal distension, and the mechanisms of the two are also different. POI in gastrectomy can often caused by the removal of motilin^6^ and damage to the vagus nerve^7^, which activates the movement of the stomach. The impaired interaction between the gut and macrophages during enterectomy is often regarded as the reason for the occurrence of POI^8^.

POI is still a frequent complication of surgery. Fluid overload, exogenous opioids, neurohormonal dysfunction, and gastrointestinal stretch and inflammation are key mechanisms in the pathophysiology of POI^9^. In our clinical practice, stellate ganglion block (SGB) will also be used to solve the problem of postoperative POI of patients as soon as possible, which has been reported in a previous meta-analysis^10^. And I hope this will give some inspiration to my surgical colleagues.

Finally, we truly thank the authors for their excellent and important work.

## Ethical approval

Not applicable.

## Consent

Not applicable.

## Sources of funding

This project was supported by the National Natural Science Foundation of China (81970459).

## Author contribution

Y.W.: literature collection and manuscript writing; S.G.: literature collection and manuscript revised; Y.P.: project development and manuscript revised.

## Conflicts of interest disclosure

All authors declare that they do not have any conflicts of interest.

## Research registration unique identifying number (UIN)

Because this is a commentary of a research paper, research registration is not required.

## Guarantor

Yisheng Pan.

## Data availability statement

This manuscript is a comment. All the data from the current study are publicly available.

## Provenance and peer review

Our paper was not invited.
